# *Mycobacterium tuberculosis* Beijing Type Mutation Frequency

**DOI:** 10.3201/eid1903.121849

**Published:** 2013-03

**Authors:** Jurriaan E.M. de Steenwinkel, Dick van Soolingen, Irma A.J.M. Bakker-Woudenberg

**Affiliations:** Author affiliations: Erasmus University Medical Center, Rotterdam, the Netherlands (J.E.M. de Steenwinkel, I.A.J.M. Bakker-Woudenberg);; National Institute of Public Health and the Environment (RIVM), Bilthoven, the Netherlands (D. van Soolingen);; Radboud University Medical Center, Nijmegen, the Netherlands (D. van Soolingen)

**Keywords:** Mycobacterium tuberculosis, drug resistance, mutation frequency, rifampin, tuberculosis and other mycobacteria, Beijing type

**In Response:** We explain the differing frequencies of mutation to rifampin resistance mentioned by Werngren (*1*). First, the strains of *Mycobacterium tuberculosis* that we tested differed from those previously tested (*2*). Second, we used different rifampin concentrations in subculture plates. For Beijing strain 2002–1585, Bergval et al. (*3*) found a mutation frequency of 4–24 × 10^−8^ at a subculture concentration of 8 mg/L, whereas we found a mutation frequency of 3–4 × 10^−3^ at a subculture concentration of 1 mg/L and a lower mutation frequency at 2 mg/L. Thus, the concentration of drugs in subculture plates is crucial to mutation frequency assays. Absent a subculture concentration standard, we applied rifampin at 1 mg/L (*4*) because bacteria growing at this concentration are considered resistant to rifampin. Our mutation frequency and time-kill kinetics assay results are not contradictory because in the time-kill kinetics assays, the subculture rifampin concentration was 4 mg/L.

We performed no classical fluctuation assays. We compared the Beijing genotype with the East African/Indian genotype to learn how *M. tuberculosis* strains differed in their capacity to withstand antituberculosis drug treatment. For reference strain H37Rv, mutation frequency was 1.5 × 10^−6^, higher than that found with higher subculture concentrations.

With regard to the 3 other issues, our drug-susceptibility testing of mutants showed a stable rifampin-resistant phenotype. We agree that these bacteria might represent preexisting mutants selected during drug exposure in a certain drug concentration window. By using different concentrations in subculture plates in our mutation frequency assay, we detected such preexisting mutants. Heteroresistance probably does not explain our observations because in our time-kill kinetics experiments, the whole mycobacterial population decreased over time in a drug concentration-dependent way, and regrowth of a drug-resistant subpopulation was not observed.

By not sticking to the fixed test conditions as used in the classical drug-susceptibility assays, research leads to highly interesting findings. One can conclude that serendipity flourishes with variation.
